# Supplementary figures and images for: The combined effects of light intensity, temperature, and water potential on wall deposition in regulating hypocotyl elongation of Brassica rapa
Source: PeerJ. 2020 May 26;8:e9106. doi: 10.7717/peerj.9106 (PMC7258941; doi:10.7717/peerj.9106)

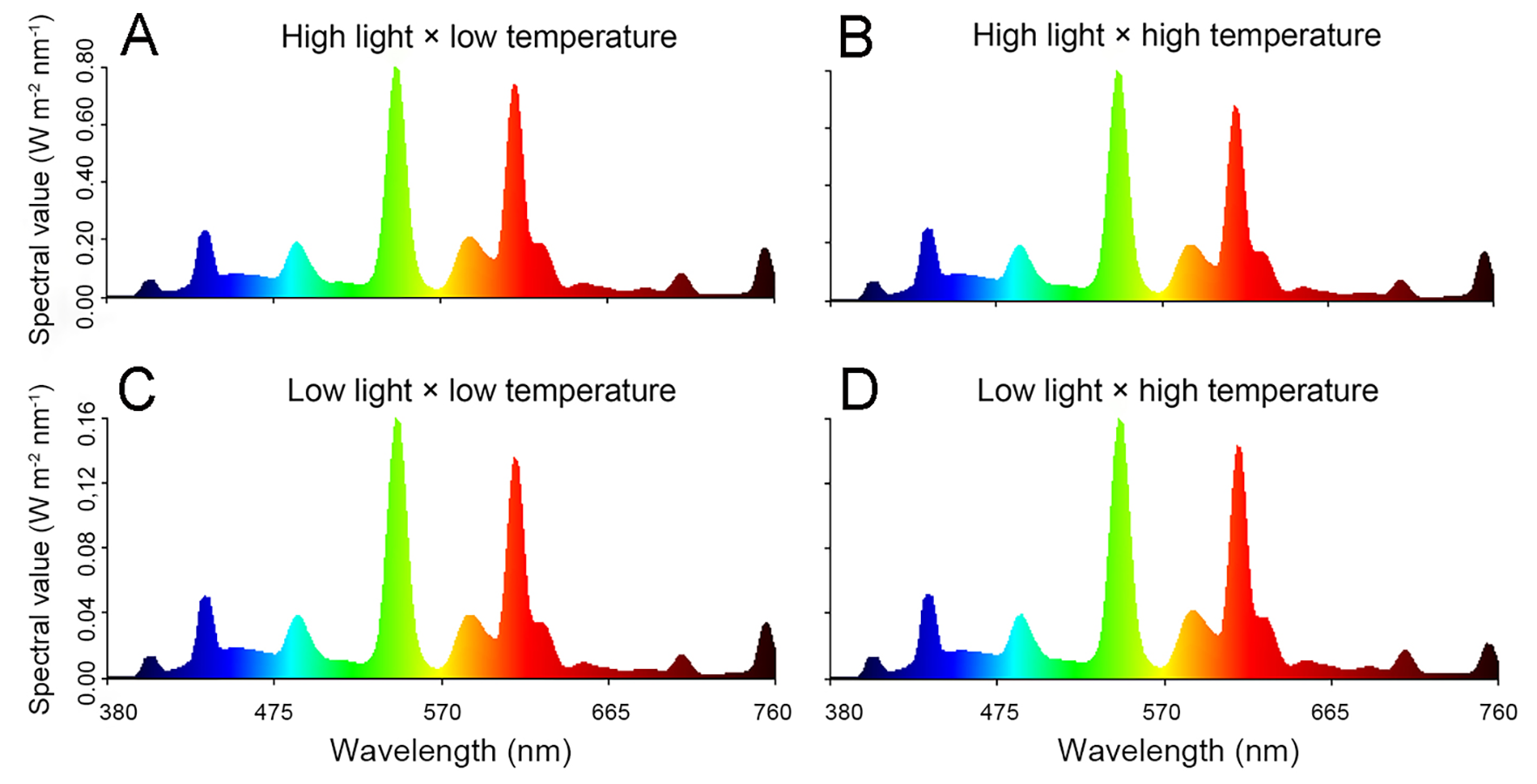

Supplement: Figure S1 — (A) Spectral output under high light × low temperature. (B) The spectral value in the condition of high light × high temperature. (C) Spectral output of low light × low temperature. (D) Spectral output in the condition of low light × high temperature. [file peerj-08-9106-s001.png]

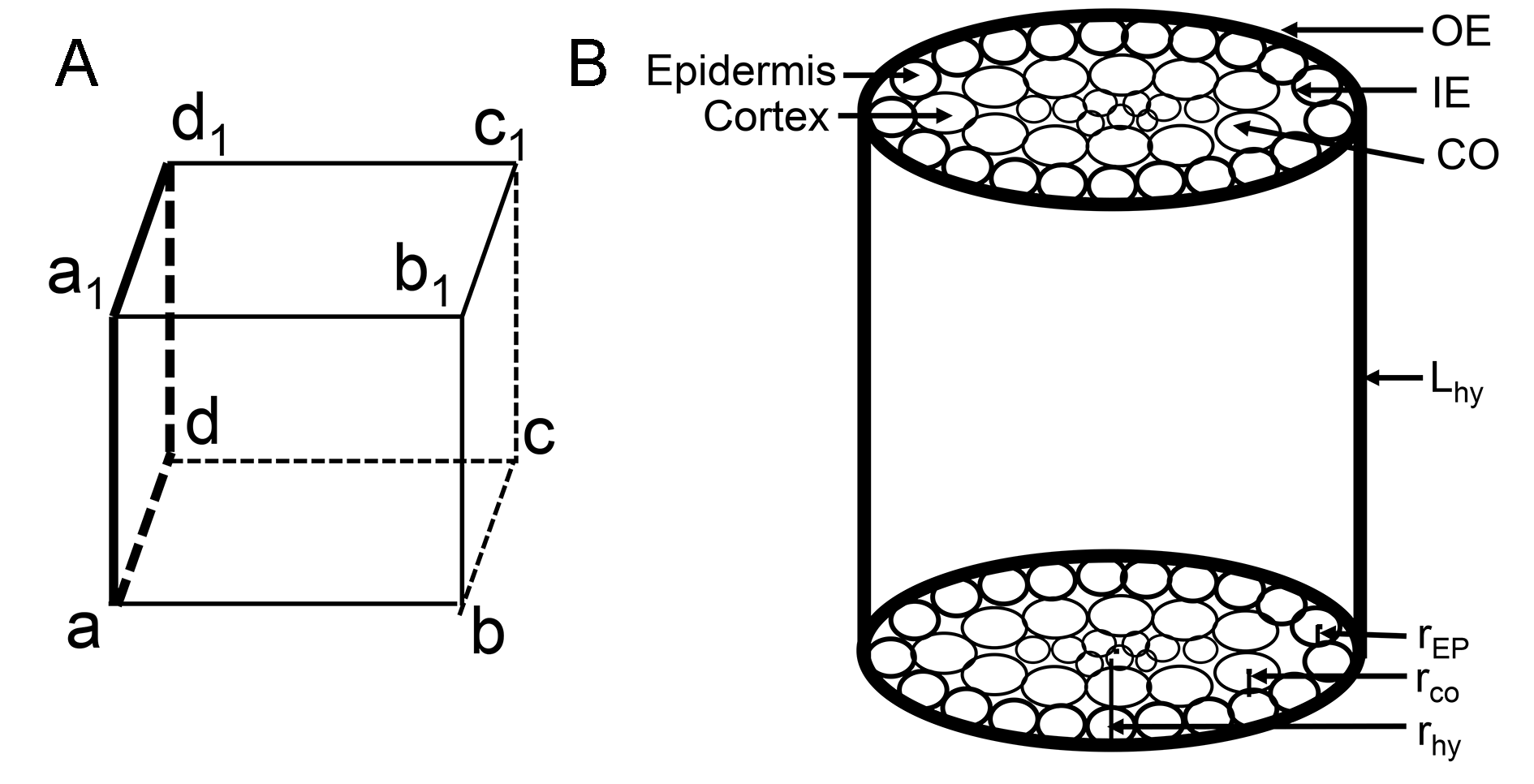

Supplement: Figure S2 — (A) The sketch map of transverse wall. Regarding the hypocotyl as a cylinder, the cube is an epidermal cell arranged in the cylinder. The two faces, perpendicular to the height of the cylinder, are the transverse wall, that is, face ABCD and face A1B1C1D1. (B) The parameters used for calculating cell wall volume. Abbreviations: OE represents outer epidermal wall; IE represents inner epidermal wall; CO represents cortical wall; Hhy represents height of hypocotyl; REP represents radius of epidermis; RCO represents radius of cortex; Rhy represents radius of hypocotyl. [file peerj-08-9106-s002.png]

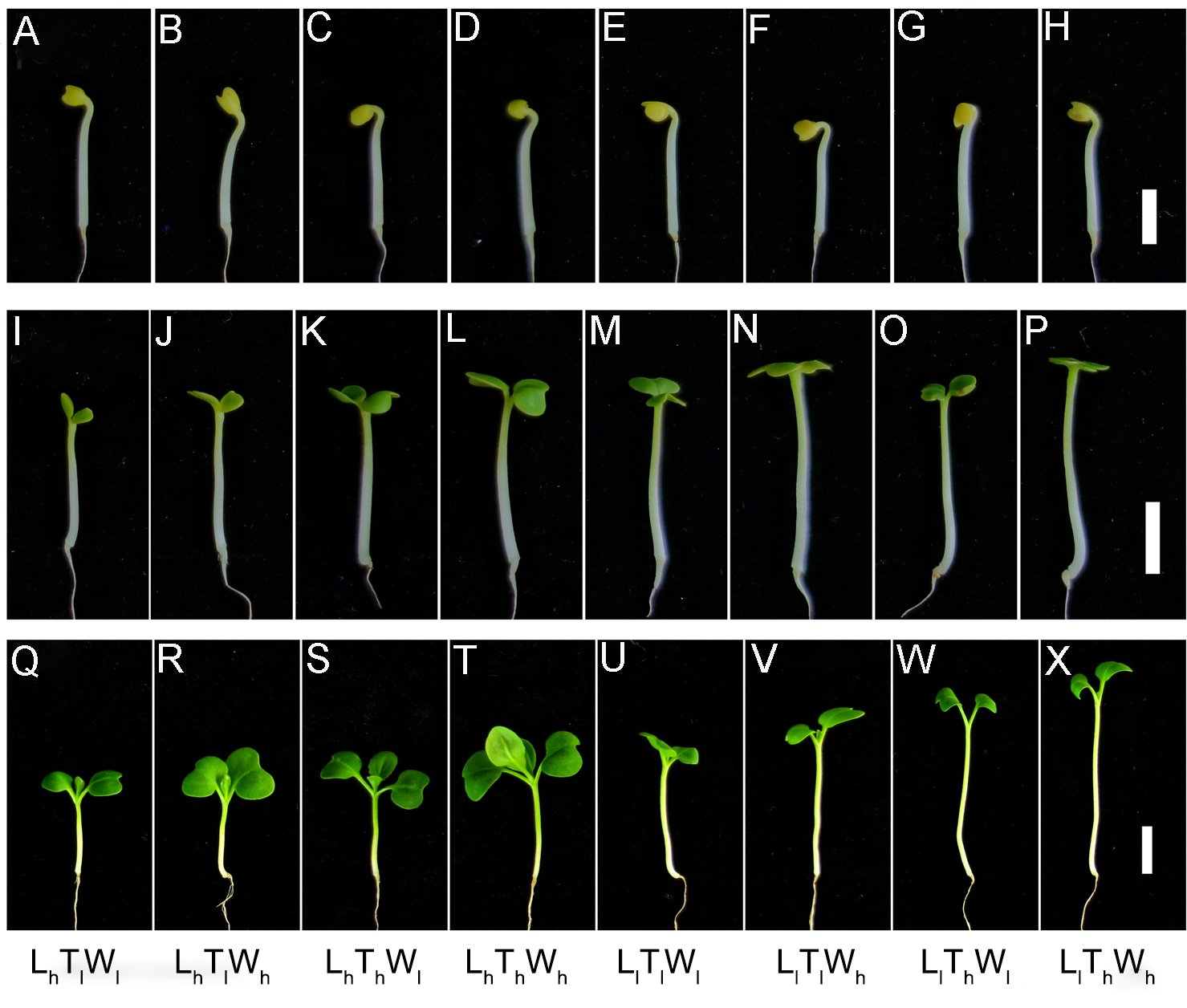

Supplement: Figure S3 — (A–H) Phenotypes of the seedlings treated for 0 d. (I–P) Morphological characteristics of seedlings treated for 2 d. (Q–X) The phenotypes of the seedlings grown for 5 d. Scale bar in A–H represents 5 mm. Scale bar in I–X represents 1 cm. Abbreviations: L, light intensity; T, temperature; W, water potential; h, high; l, low. [file peerj-08-9106-s003.png]

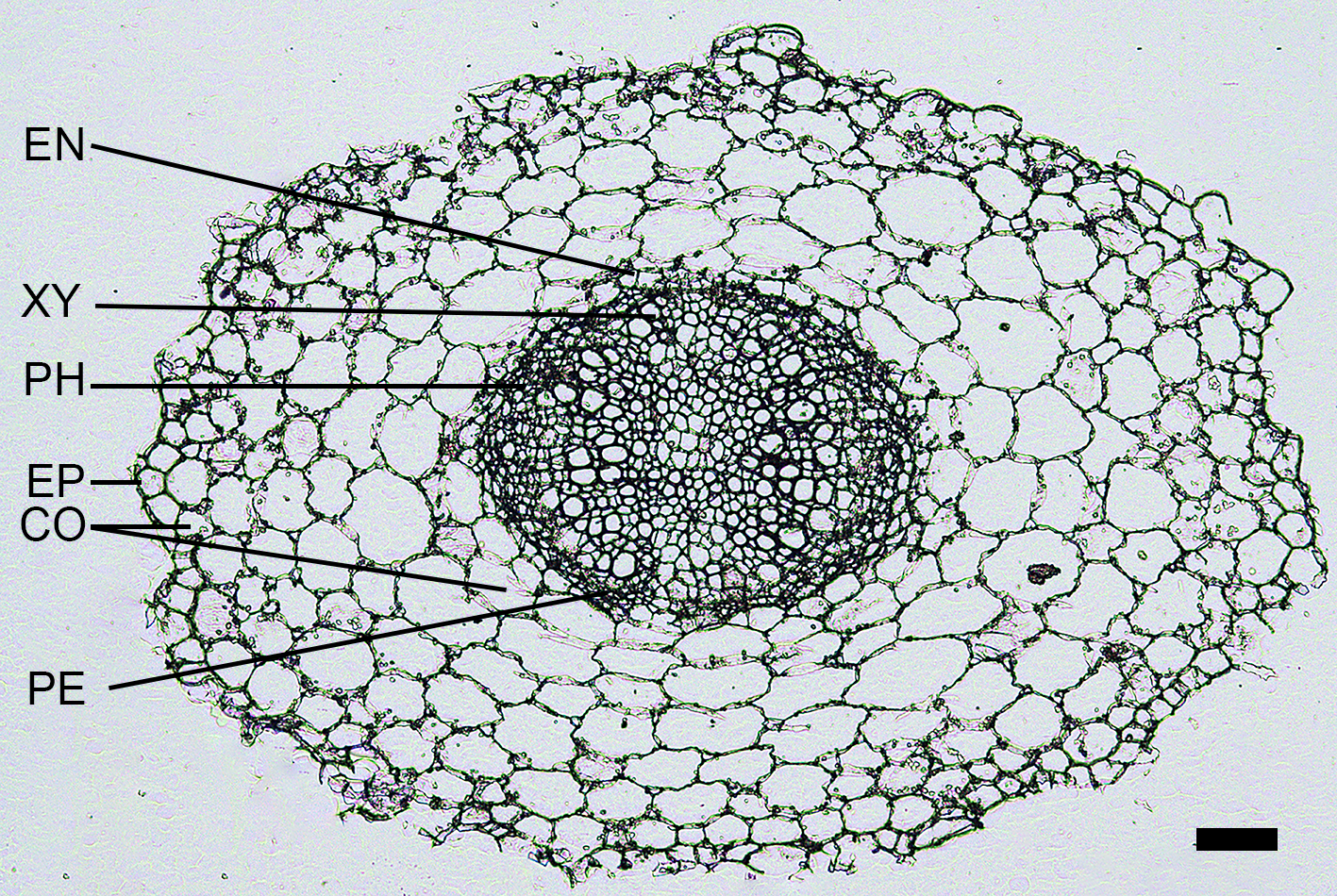

Supplement: Figure S4 — Scale bar = 200 µm. The line in the picture indicated different cell layers. Abbreviations: EP, epidermis; CT, cortex; EN, endodermis; PE, pericycle; XY, xylem; PH, phloem. [file peerj-08-9106-s004.png]

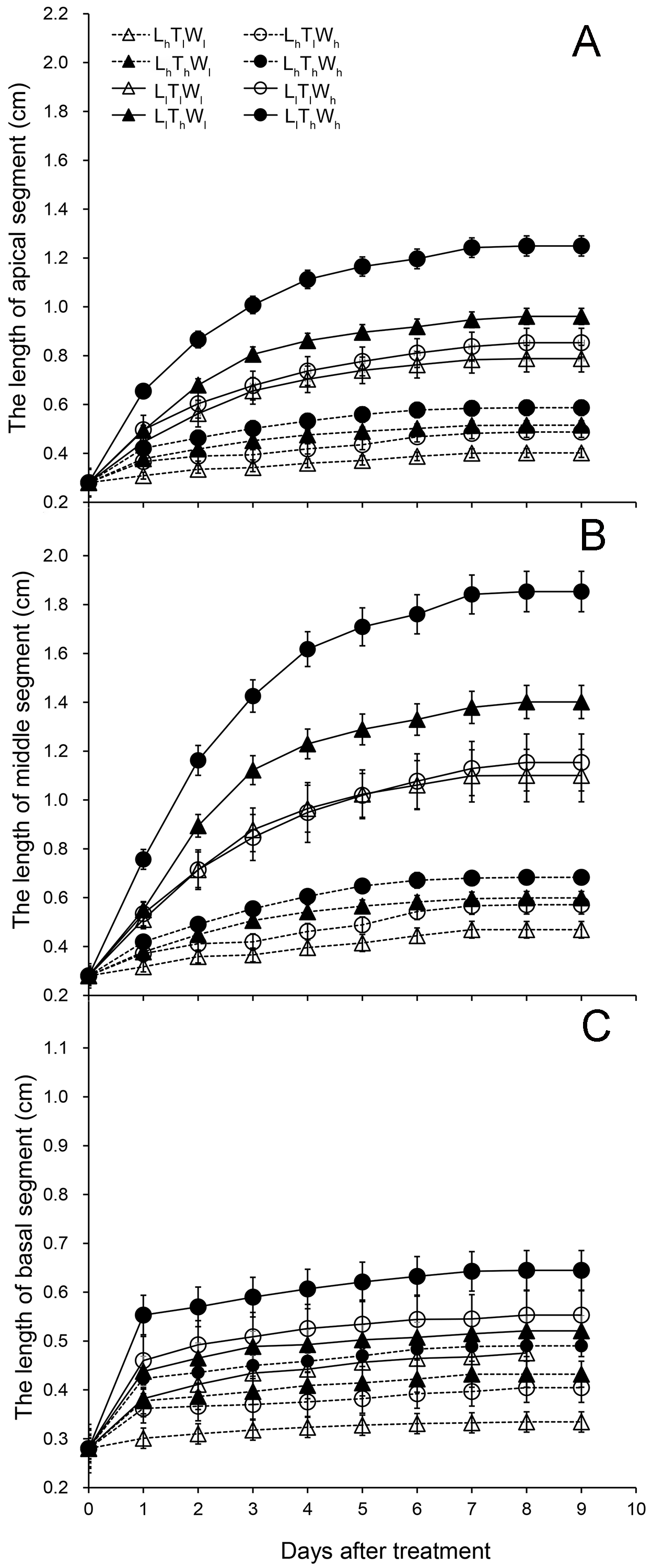

Supplement: Figure S5 — (A) Dynamic growth curve of apical segment. (B) Growth kinetics of middle segment. (C) Elongation dynamics of basal segment. Abbreviations: L, light intensity; T, temperature; W, water potential; h, high; l, low. [file peerj-08-9106-s005.png]

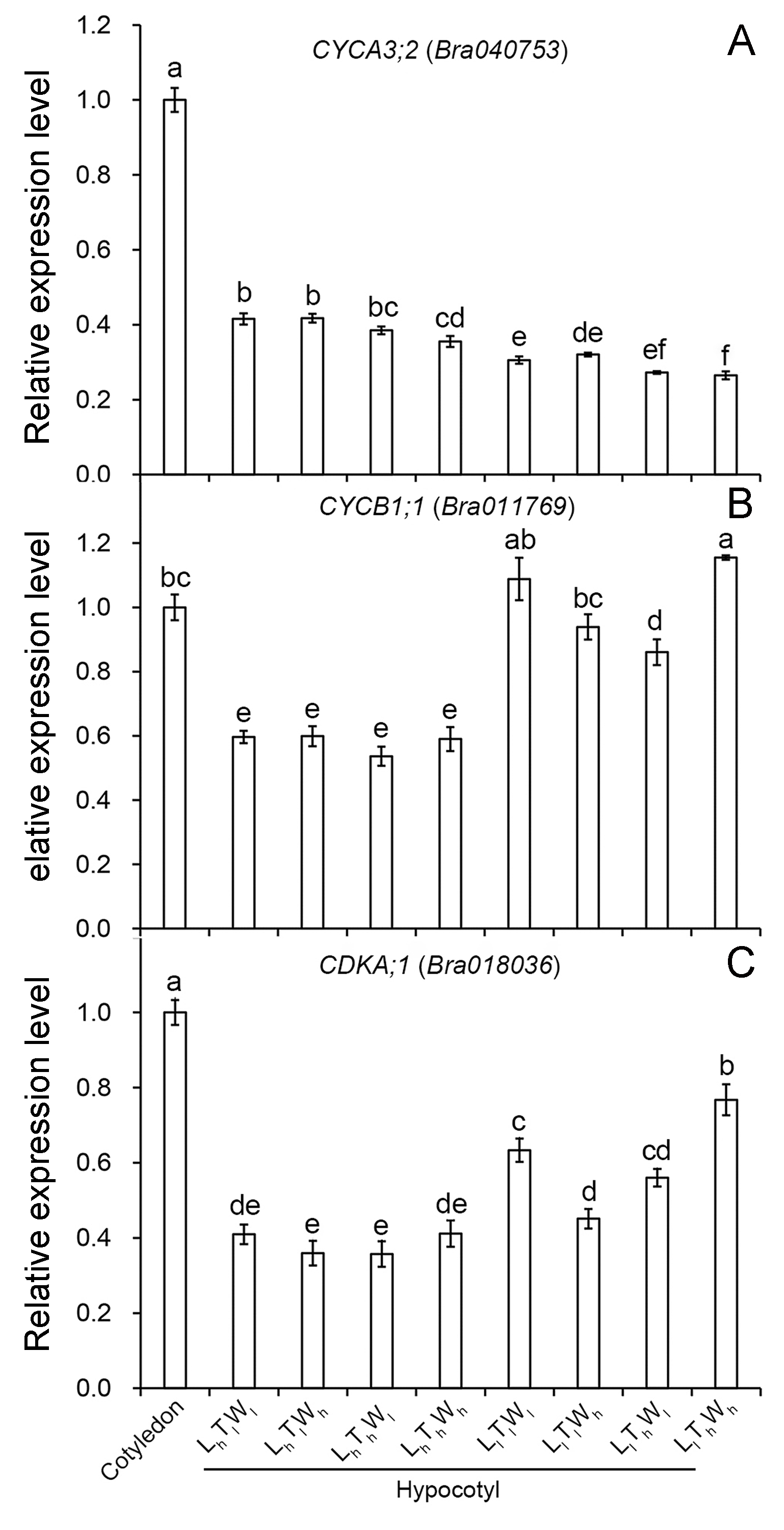

Supplement: Figure S6 — (A) Relative expression level of CYCA3;2. (B) Relative expression quantity of CYCB1;1. (C) Relative expression level of CDKA;1. The data presented in the picture are the means ± SE. Lowercase letters (a, b, c, d, e, f) represent significant differences (P < 0.05; Duncan’s multiple range test). Abbreviations: L, light intensity; T, temperature; W, water potential; h, high; l, low. [file peerj-08-9106-s006.png]

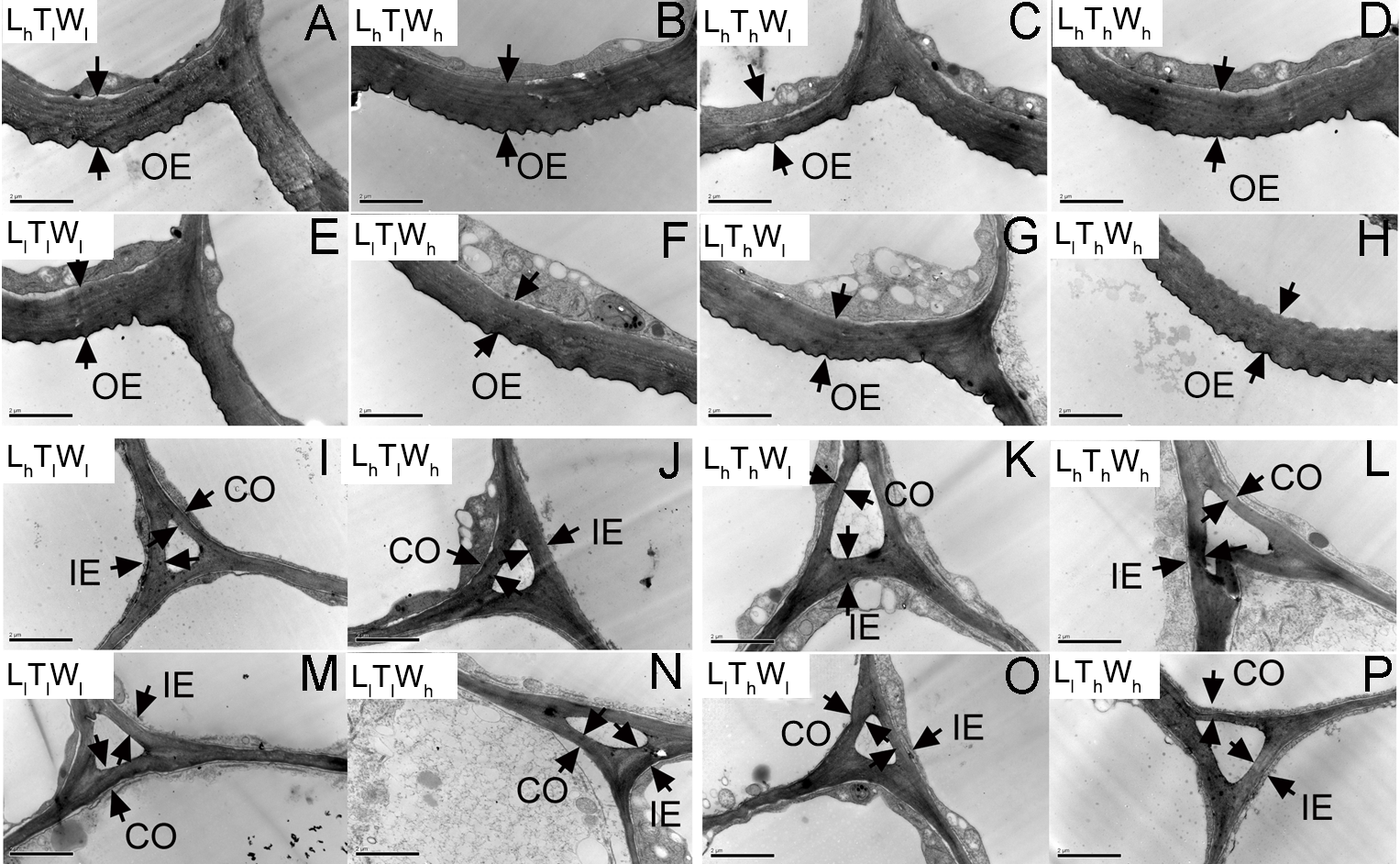

Supplement: Figure S7 — (A–H) The micrographs of OE at the mid-point in B. rapa hypocotyl. (I–P) The micrographs of the cell corner at the mid-point in B. rapa hypocotyl. Arrows in the picture indicated the walls used for thickness measurement. Abbreviations: OE, outer epidermal wall; IE, inner epidermal wall; CO, cortical wall; L, light intensity; T, temperature; W, water potential; h, high; l, low. Scale bar = 2 µm. [file peerj-08-9106-s007.png]

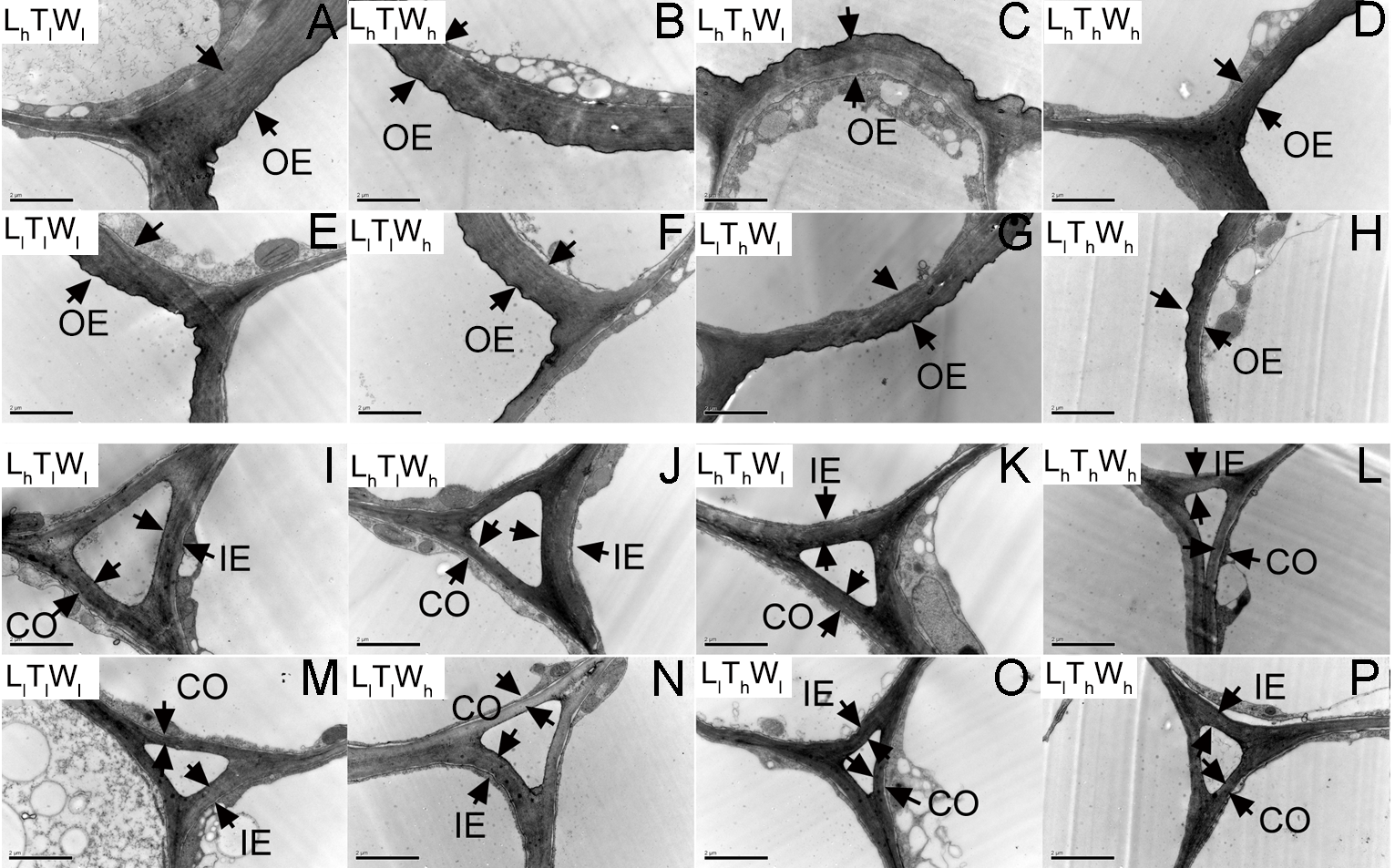

Supplement: Figure S8 — The micrographs of OE at the mid-point in B. rapa hypocotyl. (I–P) The micrographs of the cell corner at the mid-point in B. rapa hypocotyl. Arrows in the picture indicated the walls used for thickness measurement. Abbreviations: OE, outer epidermal wall; IE, inner epidermal wall; CO, cortical wall; L, light intensity; T, temperature; W, water potential; h, high; l, low. Scale bar = 2 µm. [file peerj-08-9106-s008.png]

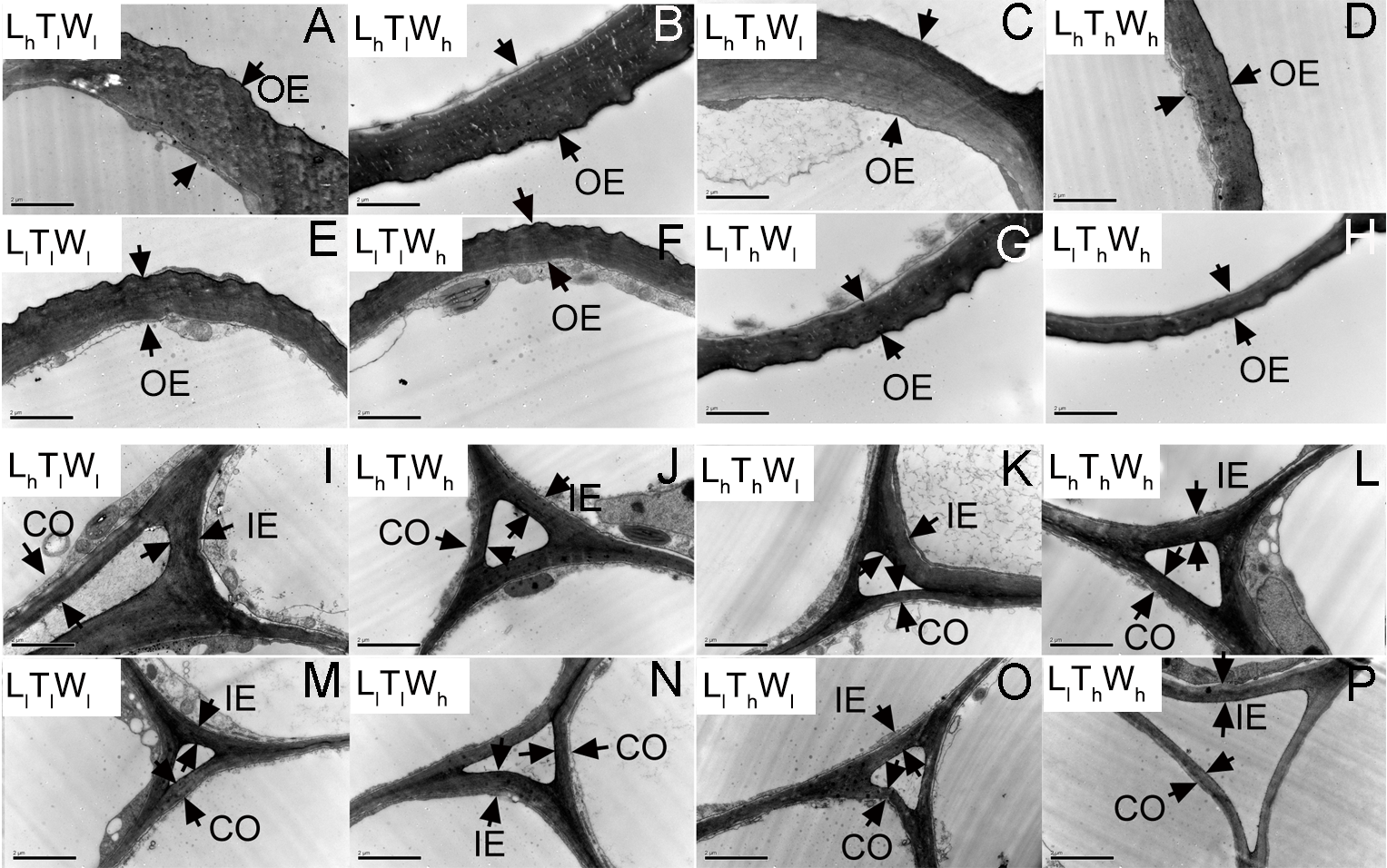

Supplement: Figure S9 — (A–H) The micrographs of OE at the mid-point in B. rapa hypocotyl. (I–P) The micrographs of the cell corner at the mid-point in B. rapa hypocotyl. Arrows in the picture indicated the walls used for thickness measurement. Abbreviations: OE, outer epidermal wall; IE, inner epidermal wall; CO, cortical wall; L, light intensity; T, temperature; W, water potential; h, high; l, low. Scale bar = 2 µm. [file peerj-08-9106-s009.png]

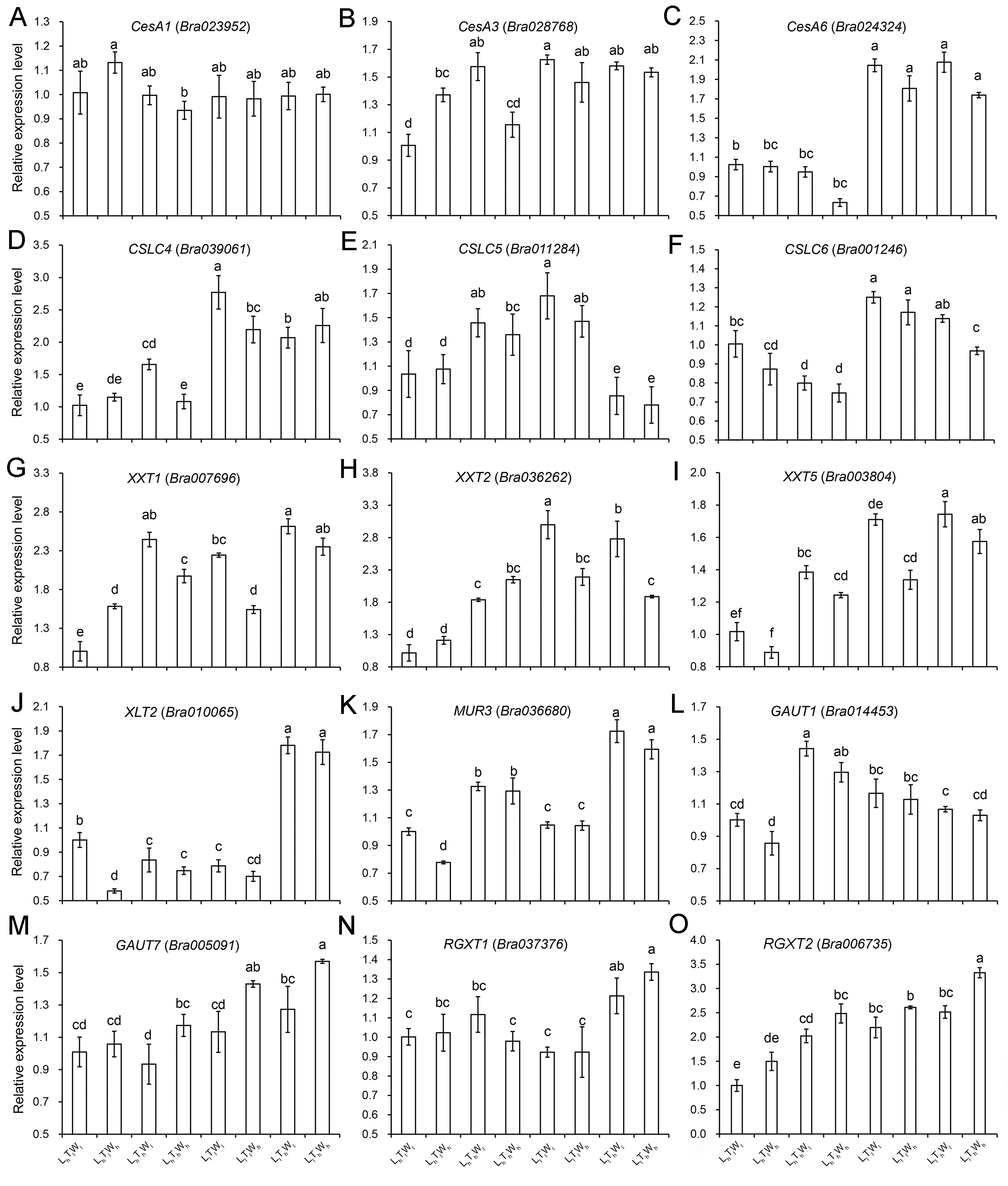

Supplement: Figure S10 — (A–O) Relative expression level of CesA1, CesA3, CesA6, CSLC4, CSLC5, CSLC6, XXT1, XXT2, XXT5, XLT2, MUR3, GAUT1, GAUT7, RGXT1, RGXT2. The data presented in the picture are the means ± SE. Lowercase letters (a, b, c, d, e, f) represent significant differences (P < 0.05; Duncan’s multiple range test). Abbreviations: L, light intensity; T, temperature; W, water potential; h, high; l, low. [file peerj-08-9106-s010.png]

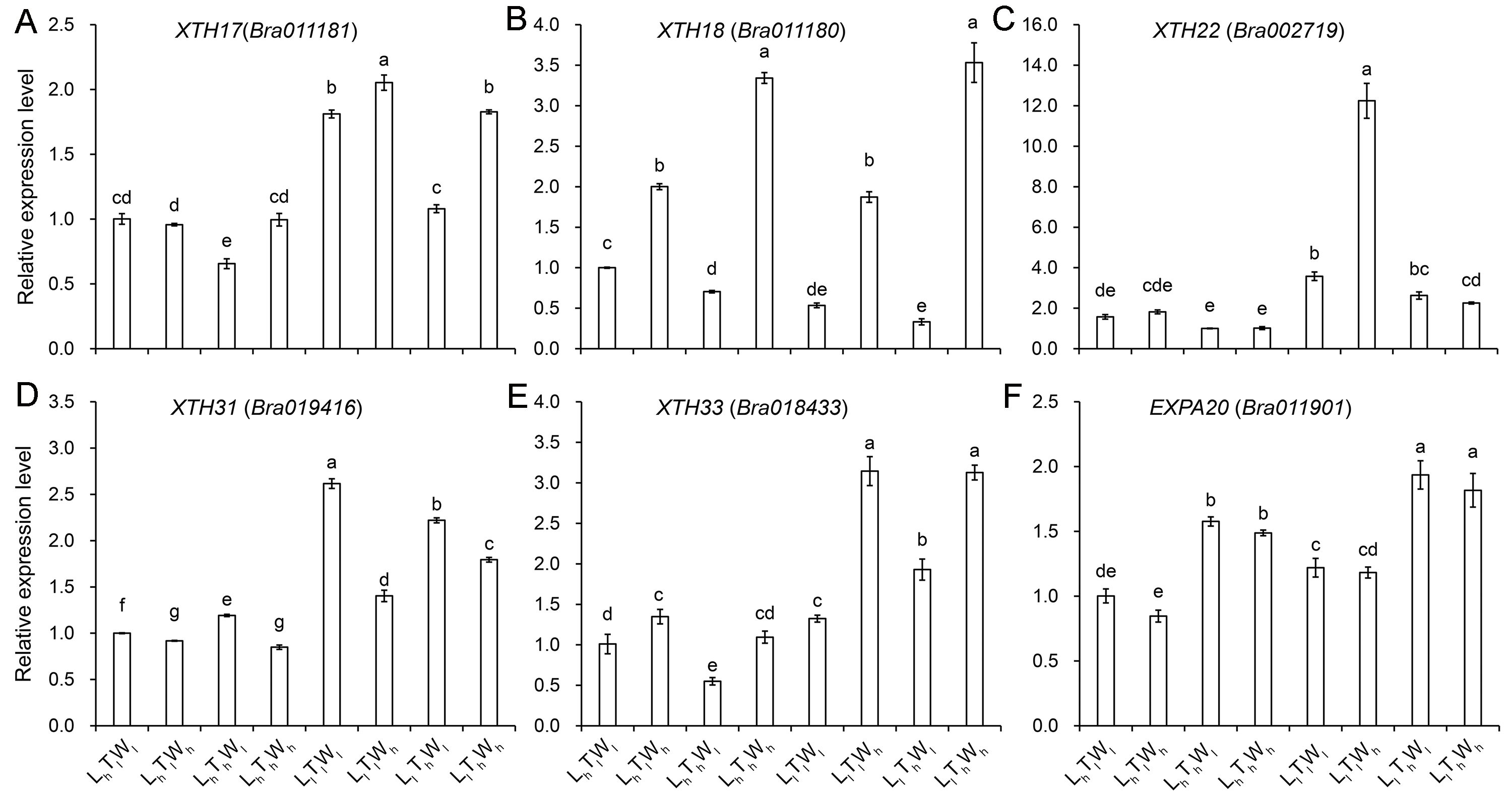

Supplement: Figure S11 — (A–F) Relative expression level of XTH17, XTH18, XTH22, XTH31, XTH33, EXPA20. The data presented in the picture are the means ± SE. Lowercase letters (a, b, c, d, e) represent significant difference (P < 0.05; Duncan’s multiple range test). Abbreviations: L, light intensity; T, temperature; W, water potential; h, high; l, low. [file peerj-08-9106-s011.png]
