# Supplementary material for: The combined effects of light intensity, temperature, and water potential on wall deposition in regulating hypocotyl elongation of Brassica rapa
Source: PeerJ. 2020 May 26;8:e9106. doi: 10.7717/peerj.9106 (PMC7258941; doi:10.7717/peerj.9106)
Supplement: Table S1 — No. represents number. [file peerj-08-9106-s012.docx]

| Gene | Accession No. | Forward primer’s sequence (5’-3’) | Reverse primer’s sequence (5’-3’) |
| --- | --- | --- | --- |
| *CesA1* | *Bra023952* | TCAGGGAGCTAACAAGGG | TGTATCAGGCGTGCGAAT |
| *CesA3* | *Bra028768* | TCTGTGGATTCCCTGTTTG | CTCCTTCTGCGGGTAGTT |
| *CesA6* | *Bra024324* | GAGGTGGTTTGAAATGGC | AGACAGATGGCAGGGAGA |
| *CSLC4* | *Bra039061* | TCACGAGGCTACAGAA | CCAGAATGCCAACGA |
| *CSLC5* | *Bra011284* | TGGCAGACGAATGGGATA | CGCAAGAGCAAACGCAAC |
| *CSLC6* | *Bra001246* | ATCTAAGGCCCAGGATTGGA | TCTGTTCCGCTGCTTATTCC |
| *XXT1* | *Bra007696* | GAAACGGCAACAACTACGA | ACGCCTCTGCTCATCCC |
| *XXT2* | *Bra036262* | GGCTACTCAGCGAGACACTT | TGCTTTAGACACCGTTCCA |
| *XXT5* | *Bra003804* | CCATTTCGTTGGGTGTAA | AGTAACCGTCTCATTCCTG |
| *MUR3* | *Bra036467* | TCAGCCTACACTCAGTACACC | TCCACCGAGACATCAAAT |
| *XLT2* | *Bra010065* | ACGTTTCTCGGCTCTGATTT | AACCGTTCCAACACTTCCTTT |
| *GAUT1* | *Bra014453* | CAACCTGAACGGTAAAGTC | ATAAGCCCATCCACAAGC |
| *GAUT7* | *Bra005091* | GCGACCTTACACTACAATGGG | TTTGCCCAGTGTAGATTCCTT |
| *RGXT1* | *Bra037376* | ACCTTCACAAGCGACCCA | TACGCCGAGGATGACGAG |
| *RGXT2* | *Bra038949* | GAAAGCAAGAGCAGCAAT | CTCCCTCGGTGTATGTCC |
| *GAPDH* | *Bra016729* | CCGCTAACTGCCTTGCTCCACTT | GCGGCTCTTCCACCTCTCCAGT |
| *XTH17* | *Bra011181* | TTCTATCTTAAAGCGCCTGGAA | GATGCAATAAGTGTGAAAGCCA |
| *XTH18* | *Bra011180* | CAAATCATCTGGTTCGGGTTTT | GTCTCCTGATCCTTGTGTGTAA |
| *XTH22* | *Bra002719* | GAGAGCTTCTCACTTTGTCTCT | TGAAGTGTGTAAGGATCTCCAC |
| *XTH31* | *Bra019416* | CCGATACGTACGTATGACAGAA | TGTGCATCCCGCTAATTTAAAG |
| *XTH33* | *Bra018433* | CGGTCAAGATTTTGCCACATTA | CAGGCATGACTTTGTATCTCGA |
| *EXPA20* | *Bra011901* | CATTTGGAACTATCTCATGCGG | GTCCCACATTGCTTATCAAGAC |
| *CYCA3;2* | *Bra040753* | TCTTGCCCTTCAGTTTGAAATG | GTGTGGCACATTGAAATCTTCT |
| *CYCB1;1* | *Bra011769* | TAAGATCAACCATCCTCGTACG | TCTTTTCTTTGGCTTTGGTACG |
| *CDKA;1* | *Bra018036* | GTCTCATCACTACTCTACACCG | ACAAAAGATTCCAAGTCCGTTG |
