# Supplementary material for: The combined effects of light intensity, temperature, and water potential on wall deposition in regulating hypocotyl elongation of Brassica rapa
Source: PeerJ. 2020 May 26;8:e9106. doi: 10.7717/peerj.9106 (PMC7258941; doi:10.7717/peerj.9106)
Supplement: Table 2 — The P value is calculated according to the Duncan’s multiple range test, revealing the effect of environmental factor on wall thickness. If the P value is less than 0.05, the effect reaches significant level. Abbreviations: L, light intensity; T, temperature; W, water potential; OE, outer epidermal wall; IE, inner epidermal wall; CO, cortical wall. [file peerj-08-9106-s013.docx]

| *P* value | | L | T | W | L × T | L × W | T× W | L × T × W |
| --- | --- | --- | --- | --- | --- | --- | --- | --- |
| OE | 2 d | < 0.0001 | < 0.0001 | < 0.0001 | < 0.0001 | 0.3119 | 0.0001 | 0.0047 |
|  | 5 d | < 0.0001 | < 0.0001 | < 0.0001 | 0.0003 | 0.0033 | 0.0015 | 0.1307 |
|  | 8 d | < 0.0001 | < 0.0001 | < 0.0001 | 0.0220 | 0.7084 | 0.0009 | < 0.0001 |
| IE | 2 d | < 0.0001 | < 0.0001 | < 0.0001 | 0.0191 | 0.0008 | 0.7949 | 0.0113 |
|  | 5 d | < 0.0001 | < 0.0001 | < 0.0001 | 0.3343 | 0.7438 | 0.0653 | 0.0013 |
|  | 8 d | < 0.0001 | < 0.0001 | < 0.0001 | 0.2508 | < 0.0001 | 0.0002 | 0.0143 |
| CO | 2 d | < 0.0001 | < 0.0001 | < 0.0001 | 0.6626 | 0.0042 | 0.1244 | 0.0733 |
|  | 5 d | < 0.0001 | < 0.0001 | < 0.0001 | 0.0313 | 0.0013 | < 0.0001 | < 0.0001 |
|  | 8 d | < 0.0001 | < 0.0001 | < 0.0001 | 0.0220 | 0.7084 | 0.0009 | < 0.0001 |
