# Supplementary material for: The combined effects of light intensity, temperature, and water potential on wall deposition in regulating hypocotyl elongation of Brassica rapa
Source: PeerJ. 2020 May 26;8:e9106. doi: 10.7717/peerj.9106 (PMC7258941; doi:10.7717/peerj.9106)
Supplement: Table S3 — The P values are calculated according to the Duncan’s multiple range test, which is used to indicate the effect of environmental factors on the content of cell wall components. When p value is less than 0.05, the effect is significant. Abbreviations: L represents light intensity; T represents temperature; W represents water potential. [file peerj-08-9106-s014.docx]

| *P* value | L | T | W | L × T | L × W | T × W | L × T × W |
| --- | --- | --- | --- | --- | --- | --- | --- |
| Wall mass | < 0.0001 | < 0.0001 | < 0.0001 | 0.2168 | 0.1956 | 0.5752 | 0.1371 |
| Cellulose | < 0.0001 | 0.7778 | 0.7020 | 0.5697 | 0.0138 | 0.8097 | 0.2013 |
| Hemicellulose | < 0.0001 | 0.3984 | 0.6534 | 0.2872 | 0.6350 | 0.7752 | 0.8638 |
| Pectin | < 0.0001 | 0.0346 | 0.0374 | 0.0020 | 0.8777 | 0.0019 | 0.0022 |
