# Supplementary material for: The combined effects of light intensity, temperature, and water potential on wall deposition in regulating hypocotyl elongation of Brassica rapa
Source: PeerJ. 2020 May 26;8:e9106. doi: 10.7717/peerj.9106 (PMC7258941; doi:10.7717/peerj.9106)
Supplement: Table S4 — The P values are calculated according to the Duncan’s multiple range test, indicating the effect of environmental factors on the expression level of target genes. The effect reaches significant level, when p value is less than 0.05. Abbreviations: L represents light intensity; T represents temperature; W represents water potential. [file peerj-08-9106-s015.docx]

| *P* value | L | T | W | L × T | L × W | T × W | L × T × W |
| --- | --- | --- | --- | --- | --- | --- | --- |
| *CesA1* (*Bra023952*) | 0.2447 | 0.7338 | 0.1624 | 0.6711 | 0.2965 | 0.2001 | 0.2447 |
| *CesA3* (*Bra028768*) | 0.1208 | 0.2624 | 0.1846 | 0.5054 | 0.0129 | 0.0018 | 0.1208 |
| *CesA6* (*Bra024324*) | 0.1571 | 0.0135 | 0.2260 | 0.4794 | 0.2408 | 0.5434 | 0.1571 |
| *CSLC4* (*Bra039061*) | 0.4621 | 0.0176 | 0.0270 | 0.4719 | 0.6808 | 0.0008 | 0.4621 |
| *CSLC5* (*Bra011284*) | 0.2203 | 0.8516 | 0.0130 | 0.9483 | 0.6928 | 0.9311 | 0.2203 |
| *CSLC6* (*Bra001246*) | 0.0008 | 0.0127 | 0.8809 | 0.7166 | 0.9488 | 0.3037 | 0.0008 |
| *XXT1* (*Bra007696*) | < 0.0001 | 0.0199 | 0.0645 | 0.0049 | 0.0777 | 0.0004 | < 0.0001 |
| *XXT2* (*Bra036262*) | 0.0415 | 0.0500 | < 0.0001 | 0.0004 | 0.3735 | 0.7515 | 0.0415 |
| *XXT5* (*Bra003804*) | < 0.0001 | 0.1511 | 0.6456 | 0.1701 | 0.0922 | 0.1046 | < 0.0001 |
| *XLT2* (*Bra010065*) | < 0.0001 | < 0.0001 | < 0.0001 | 0.0059 | 0.0059 | 0.0169 | < 0.0001 |
| *MUR3* (*Bra036680*) | < 0.0001 | 0.0196 | 0.0196 | 0.4106 | 0.7263 | 0.0502 | < 0.0001 |
| *GAUT1* (*Bra014453*) | 0.0001 | 0.0131 | < 0.0001 | 0.1123 | 0.9796 | 0.9388 | 0.0001 |
| *GAUT7* (*Bra005091*) | 0.1620 | 0.0012 | 0.2869 | 0.1880 | 0.3718 | 0.4037 | 0.1620 |
| *RGXT1* (*Br037376*) | < 0.0001 | 0.9457 | < 0.0001 | 0.0257 | 0.6350 | 0.0114 | < 0.0001 |
| *RGXT2* (*Bra067375*) | < 0.0001 | 0.0007 | 0.0733 | 0.6098 | 0.4754 | 0.4013 | < 0.0001 |
| *XTH17* (*Bra011181*) | < 0.0001 | < 0.0001 | < 0.0001 | < 0.0001 | < 0.0001 | 0.1741 | < 0.0001 |
| *XTH18* (*Bra011180*) | < 0.0001 | < 0.0001 | 0.1041 | 0.0018 | < 0.0001 | 0.3640 | < 0.0001 |
| *XTH22* (*Bra002719*) | < 0.0001 | < 0.0001 | < 0.0001 | < 0.0001 | < 0.0001 | < 0.0001 | < 0.0001 |
| *XTH31* (*Bra019416*) | 0.0833 | < 0.0001 | 0.0833 | < 0.0001 | < 0.0001 | < 0.0001 | 0.0833 |
| *XTH33* (*Bra018433*) | 0.6686 | < 0.0001 | < 0.0001 | < 0.0001 | 0.1146 | 0.0056 | 0.6686 |
| *EXPA20* (*Bra011901*) | < 0.0001 | 0.0010 | 0.1914 | 0.3873 | 0.9462 | 0.1533 | < 0.0001 |
